# A Novel Computational Framework for Precision Diagnosis and Subtype Discovery of Plant With Lesion

**DOI:** 10.3389/fpls.2021.789630

**Published:** 2022-01-03

**Authors:** Fei Xia, Xiaojun Xie, Zongqin Wang, Shichao Jin, Ke Yan, Zhiwei Ji

**Affiliations:** ^1^College of Artificial Intelligence, Nanjing Agricultural University, Nanjing, China; ^2^Center for Data Science and Intelligent Computing, Nanjing Agricultural University, Nanjing, China; ^3^Plant Phenomics Research Centre, Academy for Advanced Interdisciplinary Studies, Regional Technique Innovation Center for Wheat Production, Key Laboratory of Crop Physiology and Ecology in Southern China, Ministry of Agriculture, Nanjing Agricultural University, Nanjing, China; ^4^Collaborative Innovation Centre for Modern Crop Production co-sponsored by Province and Ministry, Jiangsu Key Laboratory for Information Agriculture, Nanjing Agricultural University, Nanjing, China; ^5^Department of Building, School of Design and Environment, National University of Singapore, Singapore, Singapore

**Keywords:** plant, disease diagnosis, subtype discovery, deep learning, t-SNE, image clustering

## Abstract

Plants are often attacked by various pathogens during their growth, which may cause environmental pollution, food shortages, or economic losses in a certain area. Integration of high throughput phenomics data and computer vision (CV) provides a great opportunity to realize plant disease diagnosis in the early stage and uncover the subtype or stage patterns in the disease progression. In this study, we proposed a novel computational framework for plant disease identification and subtype discovery through a deep-embedding image-clustering strategy, Weighted Distance Metric and the t-stochastic neighbor embedding algorithm (WDM-tSNE). To verify the effectiveness, we applied our method on four public datasets of images. The results demonstrated that the newly developed tool is capable of identifying the plant disease and further uncover the underlying subtypes associated with pathogenic resistance. In summary, the current framework provides great clustering performance for the root or leave images of diseased plants with pronounced disease spots or symptoms.

## Introduction

Plants are often attacked by various pathogens (e.g., bacteria, viruses, fungi, etc.) during their growth and development (Suzuki et al., 2014), resulting in abnormal physiological and morphological changes in plants. In severe cases, it may disrupt its normal growth and development and even cause large-scale disasters, such as leaf spot disease (Ozguven and Adem, 2019), powdery mildew (Lin et al., 2019), brown spot and blast diseases (Phadikar and Goswami, 2016), and gray mold (Fahrentrapp et al., 2019). The prior symptoms of these diseases include leaf discoloration, tissue deformation or necrosis, and root atrophy, etc. Plant diseases, especially crop diseases, may cause social problems such as economic losses or food shortages in a certain area (Wilkinson et al., 2011). Therefore, early diagnosis of plant diseases, especially the precise prediction of plant disease severity and drug resistance (Bock et al., 2020), will help formulate effective control strategies, thereby effectively prevent the spread of diseases and reduce economic losses (Liang et al., 2019). To solve the above problems, many researchers made great efforts on the diagnosis of plant diseases by exploring the relationship between pathogen infection and plant disease symptoms (Bass et al., 2019; Vishnoi et al., 2021). However, these studies cannot provide real-time disease diagnosis and even evolution trajectory inference and will cause delays or misjudgments in decision-making. In recent years, plant phenomics (Tardieu et al., 2017; Pasala and Pandey, 2020) was generated, which can automatically and non-destructively obtain high-throughput plant phenotyping images (Lee et al., 2018; Li et al., 2020), which makes computer-aided rapid diagnosis and real-time monitoring of plant diseases possible.

Computationally, phenomics-based plant disease diagnosis can be grouped into two categories, one is *semantic* feature-based models, and the other is *non-sematic* feature-based models (e.g., deep learning [DL] models). The first category (conventional image processing) is characterized by the features of color (Gaikwad and Musande, 2017), texture (Hossain et al., 2019; Ismail et al., 2020), and shape (Chouhan et al., 2020) extracted from the lesion area of the phenotypic images to achieve disease diagnosis and prediction. For example, Zhang et al. (2017) segmented the lesions from the leaf images and extracted the shape and color features for disease recognition in cucumber. Moreover, some researchers realized the automatic diagnosis of plant diseases through a classifier built with texture features (Hossain et al., 2019; Ismail et al., 2020). In addition, computer vision (CV) and machine learning were applied to quantify root traits in real time for precision plant breeding (Rahaman et al., 2019; Falk et al., 2020). However, the variation of plant phenomics and the dependence of prior knowledge always limit the generalization of this type of method to different plant diseases. In recent years, DL has been widely used in image classification and clustering (Hu et al., 2020; Saleem et al., 2020). The representative characterizations of DL-based models include powerful capabilities for feature extraction, low dependence on domain knowledge, and high predictive accuracy (Too et al., 2019; Lee et al., 2020). In the past few years, DL was used to analyze the phenomics of plant disease. Various convolutional neural network (CNN) models were developed as the image multi-class classifiers to distinguish different plant leaf diseases from high-throughput phenomics (Brahimi et al., 2018; Zhang et al., 2019). Furthermore, DL is also very effective for grading the severity of plants with the same disease (Verma et al., 2020). Liang et al. (2019) combined ResNet50 (Wen et al., 2020) model and Shufflenet-V2 (Ghosh et al., 2020) to build a PD^2^SE-Net network model, which realized the classification of plant diseases and the prediction of disease severity. Yu et al. (2006) applied VGG16 model on diseased leaf images for grading the severity of apple black rot (Wang et al., 2017). Although DL models are widely studied for plant disease diagnosis, they still face obvious challenges, such as poor generalization, unexplainable features, and high dependence on abundant training samples.

In this study, we proposed a novel image clustering method for both plant disease classification and subtype discovery. Firstly, all the original plant images were preprocessed to amplify the sample size. Secondly, we established a deep CNN to extract the features of phenotypic images. Finally, we designed a clustering strategy by integrating a Weighted Distance Metric (WDM) and the t-stochastic neighbor embedding algorithm, named “WDM-tSNE.” To validate the effectiveness, we applied the proposed method on a batch of public plant image datasets, namely, Modified National Institute of Standards and Technology (MNIST) (Deng, 2012), Aphanomyces Root Rot (ARR) in lentil (Marzougui et al., 2019), cherry powdery mildew, strawberry leaf scorch disease, and three types of tomato disease from *PlantVillage* dataset (Mohanty et al., 2016). The experimental results show that our method obtained high performance on plant disease classification and subtype discovery. In particular, the WDM-tSNE strategy provides better clustering accuracy than the standard tSNE.

## Related Work

In this section, we briefly review the related work of plant disease diagnosis on semantic feature-based models, and non-sematic feature-based models.

### Semantic Feature-Based Models

The general idea of this kind of method includes four steps: (1) image preprocessing; (2) lesion segmentation; (3) image features are defined and extracted for describing the pathology signatures of the lesion regions; and (4) the image samples are classified by using a machine-learning model (Vishnoi et al., 2021). Considering the fact that the accuracy of lesion segmentation directly affects the sample classification, many researchers used various image-segmentation strategies to achieve the extraction of the target regions, such as threshold-based segmentation methods (Tete and Kamlu, 2017), edge detection algorithms (Wang et al., 2018), and spatial clustering methods (Guan et al., 2017). After obtaining the lesion regions, researchers often define the color, texture, or shape features to characterize the disease state of each sample. Gaikwad and coworkers applied K-means to segment the lesion regions in the wheat leaf images and extracted the color features, such as color histogram (Stricker, 1994), color moments (Poonam and Jadhav, 2015), and the texture features [e.g., gray-Level co-occurrence matrix [GLCM] (Gadelmawla, 2004)] to construct a support-vector machine (SVM) model for the classification of wheat diseases (Gaikwad and Musande, 2017). Ali et al. (2017) applied Delta E (Δ*E*) segmentation to process the leave images of diseased potatoes and extract color and texture features based on red, green, and blue (RGB), hue, saturation, value (HSV), and local binary patterns (LBP) to implement the classification of early blight and late blight (Ismail et al., 2020). Ayyub and Manjramkar (2019) successfully classified the apple fruit diseases via a multi-class model by integrating improved sum and difference histogram (ISADH), completed local binary pattern (CLBP), and other color and texture features.

In general, this kind of method may obtain human-interpretable features and thus provide good performance on some plant diseases. However, three drawbacks exist. First, the calculation procedure of these methods is complicated. Second, these methods are highly dependent on expert knowledge. Third, they do not work well for real-time detection.

### Non-sematic Feature-Based Models

In recent years, DL has promoted the development of CV, thereby providing new ideas for image analysis and automatic diagnosis of plant diseases. In particular, the CNN model has been widely studied by researchers because of its powerful image processing and feature extraction capabilities and without the prior knowledge of domain experts (Syed-Ab-Rahman et al., 2021). At present, most of the existing works applied CNN, combined with transfer learning (Too et al., 2019) to implement plant disease diagnosis. Zhang et al. (2018) used two improved CNN models, GoogleNet and Cifar10, to classify nine types of corn diseases and obtain high accuracy. To reduce the number of parameters, Rahman et al. (2020) constructed a two-stage light CNN framework Simple-CNN to identify rice diseases with high accuracy. Moreover, other researchers made great efforts to develop novel computational models for predicting the severity of plant disease. For example, José et al. (2020) used five types of CNN models (AlexNet, GoogleNet, VGG16, ResNet50, and MobileNetV2) to estimate the severity of coffee leaf biotic stress. In addition, deep learning was also widely used to identify the diseases of fruit, root, and stem. Tan et al. (2016) presented a CNN model to recognize lesion images of diseased apples, such as scab skin, black rot, scar skin, and ring spot (Wenxue Tan, 2020). Nikhitha et al. (2019) used the Inception v3 model to detect the grades of infections in fruits (e.g., apple, banana, and cherry, *etc*.) based on color, size, and shape of the fruit (Nikhitha et al., 2019). Tusubira et al. (2020) achieved the automated scoring for root necrosis of diseased cassava by using deep CNN with semantic segmentation, which is done by classifying the necrotized and non-necrotized pixels of cassava root cross-sections without any additional feature engineering. Compared with the first category, DL models achieve higher recognition accuracy. However, we identify three limitations. First, they require large amounts of labeled data; second, they are overly sensitive to changes in the image; and third, the non-semantic features are hard to be explained.

To address the above limitations, we proposed an efficient pipeline for both disease diagnosis and severity estimation of plants with the lesion. A DL model combined with a novel clustering strategy contributes to higher prediction accuracy and lower computational cost.

## Materials and Methods

**FIGURE 1:**
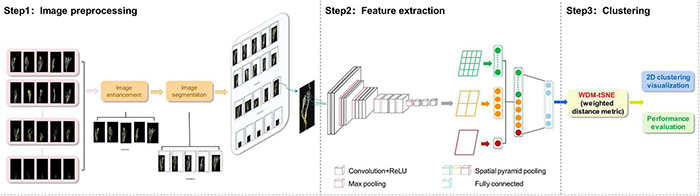
The flowchart of the proposed framework. ReLU, Rectified Linear Unit.

The proposed computational framework includes three steps (Figure 1) and will be explained in detail in the following subsections.

### Image Preprocessing

Before extracting features, each image needs to be preprocessed, such as image enhancement and image segmentation. Image augmentation is to increase the diversity of samples (Halevy et al., 2009). we use horizontal flip (Connor Shorten, 2019) and affine transformation (Shen et al., 2019) on each image to enhance the size and quality of training datasets so that better DL models can be built. The purpose of image segmentation is to obtain areas related to plant tissues (root or leaf) from the original images. Therefore, the irrelevant region needs to be removed. In this study, we detected the relevant area by traversing all the pixels in each image and obtained the smallest circumscribed rectangle (Yu et al., 2006) of the outer contour of a plant tissue.

### Feature Extraction

**FIGURE 2:**
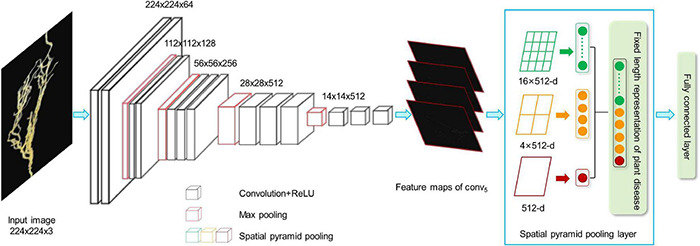
CNN-based network for feature extraction. ReLU, Rectified Linear Unit; CNN, convolutional neural network.

We developed a CNN model to extract the features from the plant images with the disease. The whole CNN model includes three layers: convolution layers, the spatial pyramid pooling (SPP) layer, and fully connected layer. The extracted high-dimensional features were further used to cluster the images with different severity levels. Figure 2 shows the details of the feature extraction process using the lentil images as an example.

#### Creating the Feature Maps

As shown in Figure 2, the first step is to create the feature maps from each input image by using a series of convolutional, non-linear, and pooling. The convolutional layers can learn the low-level features, such as edges and curves, which provide the CNN with the important property of “translation invariance” (Kayhan and van Gemert, 2020). That makes it unnecessary to focus on the location of the disease on the plant roots or leaves and let alone to divide up the area of the spot. Convolution is done by applying filters to the input image data, which decreases its size (Yamashita et al., 2018). An additional operation called the Rectified Linear Unit (ReLU) (Atila and Sengür, 2021) was used after every convolution operation to generate a non-linear relationship between input and output. Finally, The pooling layer is used for secondary feature extraction, retaining the main features, reducing parameters, saving computing resources, preventing over-fitting, and improving model generalization (Suarez-Paniagua and Segura-Bedmar, 2018). Here, we define a spatial neighborhood with a 2 × 2 window and take the largest element from the rectified feature map within that window. Max pooling not only reduces the dimensionality of each feature map but also retains the most important information. Comparing with the typical VGG16 model (Qassim et al., 2018), the network structure of our model retains all the convolutional and pooling layers and the activation method, but removes three fully connected layers.

Let us say we have a plant image, and its size is 224 × 224. The representative array of this image will be 224 × 224 × 3 (3 refers to the channels of RGB). After the first operation of convolution, we obtained the feature maps as an array with 224 × 224 ×64. Passing this array through four convolutional layers, we finally obtained 512 feature maps with 14 × 14. The final output feature map (14 × 14 × 512) will be converted into one-dimensional vector.

Considering the fact that a CNN model may take time to train on large datasets, transfer learning (Pan and Yang, 2010) was considered in our study to re-use the model weights from pre-trained ImageNet (Krizhevsky et al., 2012) tasks. Here, we directly use the five convolutional layers from the entire architecture of the pre-trained the VGG16 model on ImageNet datasets.

#### Converting the Feature Maps to a Fixed Length Feature Vector

In this step, we convert all the two-dimensional feature maps to a single long continuous linear vector because the fully connected layer expects to receive one-dimensional inputs (Gu et al., 2018). Here, we introduce SPP (He et al., 2015) layer to remove the limitation of the fixed size of the images. The SPP layer was placed after the last convolutional layer and aggregated multi-scale features. As shown in Figure 2, each feature map (14 × 14) is divided into a lattice of n × n (*n* = 1,2,4) and each lattice is pooled, resulting in 21 features. This also means that the 512 feature maps of an original image are finally represented as a one-dimensional vector with a length of 10,752 (21 × 512). The output of the fully connected layer is 4,096, which means each image matrix will be converted to a feature vector with length 4,096 for clustering calculation.

### Image Clustering

As mentioned above, each original image was finally represented as a 4,096 × 1 vector after the feature extraction process. The clustering of a group of original images is thus equivalent to a clustering task on a set of data points with a dimension of 4,096. Considering the fact that t-SNE is an efficient algorithm based on manifold learning for unsupervised clustering (Van der Maaten and Hinton, 2008), we designed an improved t-SNE algorithm for image clustering to classify plant diseases and graded the severity of a disease. The standard t-SNE algorithm assumes that the samples are distributed on a statistical manifold and converts the Euclidean distance between the samples into conditional probabilities to characterize the similarity between the samples (Talwalkar et al., 2008). However, the variables in the high-dimensional space often present complex non-linear relationships, and the Euclidean distance does not well reflect the real distribution of the samples, thus affecting its projection to the low-dimensional space. Within a manifold space, the Euclidean distance metrics can only represent the real distance between samples in a very small neighborhood subspace (Zhang et al., 2011).

Taken above together, we think that only the data points in the local neighborhood are applicable to the Euclidean distance, and they should be given greater weight in the conditional probability transformation. In this study, we adopted a WDM strategy to improve the t-SNE algorithm (WDM-tSNE) so that the similarity between samples can be better reflected after they are projected to a low-dimensional space. The details of WDM-tSNE are described as follows:

Firstly, we construct the distance matrix *D* of all the samples, where the element *d*_*ij*_ represents the distance between any two points *X*_*i*_ and *X*_*j*_ [Eq. (1)]:


(1) $$d_{ij}=\sum_{k=0}^{n}{\left(X_{ik}-X_{jk}\right)}^{2}$$

All the non-zero elements *d*_*ij*_ (*i*≠*j*) are sorted in ascending order, and the distance value that ranks approximately 10% is selected as the threshold of the neighborhood relationship, denoted asθ. If *d*_*ij*_≤θ, *X*_*i*_ and *X*_*j*_ have a neighbor relationship and weighting their distance will make them closer in the low-dimensional space. Therefore, we define a WDM strategy to adjust the distance coefficient *l* between any pair of samples *X*_*i*_ and *X*_*j*_:


(2) $$l=\{\frac{d_{ij}-d_{\min}+c}{\begin{array}{c} d_{\max}-d_{\min}\\ 1,otherwise \end{array}},ifd_{ij}\leq \theta$$

Under the Gaussian distribution centered on the point *X*_*i*_, the conditional probability *P*_*j|i*_ is used to measure the similarity between *X*_*i*_ and *X*_*j*_. In other words, *P*_*j|i*_means the probability that *X*_*i*_ chooses *X*_*j*_ as its neighbor. We thus construct conditional probability *P*_*j|i*_ for *X*_*i*_ and *X*_*j*_, and the probability distribution is defined as Eq. (3):


(3) $$P_{j|i}=\frac{\exp\left(-l*{||X_{i}-X_{j}||}^{2}/2\sigma_{i}^{2}\right)}{\sum_{k\neq i}\exp\left(-l*{||X_{i}-X_{k}||}^{2}/2\sigma_{i}^{2}\right)}$$

From Eq. (3), we have ***P*_*i*|*i*_ = 0**. Assuming that the points *Y*_*i*_ and *Y*_*j*_ in the low-dimensional space are projected from *X*_*i*_ and*X*_*j*_, the similarity between the points *Y*_*i*_ and *Y*_*j*_ can be defined as:


(4) $$Q_{j|i}=\frac{\exp\left(-{||Y_{i}-Y_{j}||}^{2}\right)}{\sum_{k\neq i}\exp\left(-{||Y_{i}-Y_{k}||}^{2}\right)}$$

According to the above description, we expect that if two points are similar in the high-dimensional space, they should be closer after being projected to the low-dimensional space. Here, we use Kullback-Leibler divergence (Van der Maaten and Hinton, 2008) to measure the difference between the above two conditional probability distributions and define the following objective function as Eq. (5):


(5) $$C=\sum_{i}KL\left(P||Q_{i}\right)=\sum_{i}\sum_{j}P_{j|i}\log\frac{p_{j|i}}{Q_{j|i}}$$

However, the KL divergence (Kullback-Leibler divergence) is asymmetric [*KL*(*P*||*Q*) ≠ *KL*(*Q*||*P*)] (Afgani et al., 2008), which will cause the gradient calculation to be complicated. To optimize the KL divergence in SNE, t-SNE adopts symmetric SNE, that is, assuming *P*_*j*|*i*_ = *P*_*i*|*j*_ and *Q*_*j*|*i*_ = *Q*_*i*|*j*_. The conditional probability *p_j|i_* can be replaced with the joint probability *p*_*ij*_:


(6) $$p_{ij}=\frac{\exp\left(-l*{||X_{i}-X_{j}||}^{2}/2\sigma^{2}\right)}{\sum_{k\neq S}\exp\left(-l*{||X_{k}-X_{S}||}^{2}/2\sigma^{2}\right)}$$

If *X*_*i*_ is an abnormal point, all the *d*_*ij*_ will be very large and may impact the calculation of *P*_*ij*_. Therefore, we define the joint probability distribution *P*_*ij*_ as:


(7) $$P_{ij}=\frac{p_{j|i}+p_{i|j}}{2n}$$

To make the points in the same cluster in the low-dimensional space more closer and the points in different clusters are more distant (Van der Maaten and Hinton, 2008), the long-tailed t-distribution is used instead of the Gaussian distribution. The joint probability of two points in the low-dimensional space can be defined as:


(8) $$Q_{ij}=\frac{\left(1+{||y_{i}-y_{j}||}^{-1}\right)}{\sum_{k\neq S}{\left(1+{||y_{k}-y_{s}||}^{2}\right)}^{-2}}$$

Therefore, Eq. (5) can be written as Eq. (9):


(9) $$C=KL\left(P||Q\right)=\sum_{i}\sum_{j}P_{ij}\log_{Q_{ij}}^{p_{ij}}$$

The formula (9) can be optimized by using the gradient descent strategy shown in formula (10):


(10) $$\frac{\delta c}{\delta y_{i}}=4\sum_{j}\left(P_{ij}-Q_{ij}\right)\left(Y_{i}-Y_{j}\right){\left(1+{||Y_{i}-Y_{j}||}^{2}\right)}^{-1}$$

Finally, all the point pairs of *X*_*i*_ and *X*_*j*_ in the high-dimensional space are projected to the two-dimensional space as *Y*_*i*_ and *Y*_*j*_. The visualization of all the points *Y* can show the clustering effect of image samples.

### Experimental Protocol

In this section, we introduced the experimental protocol designed for the validation of the proposed approach, such as data collection, simulation design, evaluation metric, and parameter optimization.

### Data Collection

The MNIST database (Modified National Institute of Standards and Technology database) (Baldominos et al., 2019), a large database of handwritten digits, was used for data collection, which is not only used for training various image processing systems but also for testing machine-learning algorithms (Pastor-López et al., 2021). Currently, the MNIST database contains 60,000 training images and 10,000 testing images. In this study, we selected a data-subset Scikit-learn containing 1,797 8 × 8 digital images to test our proposed approach for image clustering.

**FIGURE 3:**
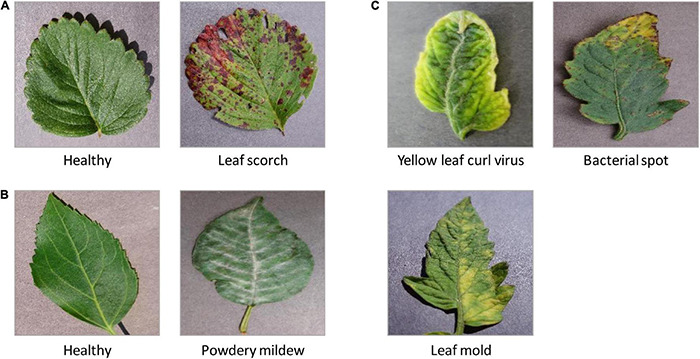
The representative leaf images with diseases from PlantVillage. **(A)** Leaf scorch of strawberry; **(B)** cherry powdery mildew; **(C)** three types of leaf diseases on tomatoes: a bacterial spot of tomato, tomato leaf mold, and tomato yellow leaf curl virus (TYLCV).

*PlantVillage* (Barbedo, 2019) is a large, open-access image database. Currently, it stores 54,306 leaf images, associate with 26 plant diseases of 14 species (Albert et al., 2017; Brahimi et al., 2017; Ferentinos, 2018). This dataset is widely employed to test the performance of machine-learning models (Wang et al., 2017). In this study, we mainly focused on the following image sets from PlantVillage: (1) three types of leaf diseases on tomatoes (Figure 3C), such as bacterial spot of tomato (Adhikari et al., 2020), tomato leaf mold (Rivas and Thomas, 2005), and tomato yellow leaf curl virus (TYLCV) (Prasad et al., 2020); (2) cherry powdery mildew (Gupta et al., 2017; Figure 3B); (3) leaf scorch of strawberry (Dhanvantari, 1967; Figure 3A).

**FIGURE 4:**
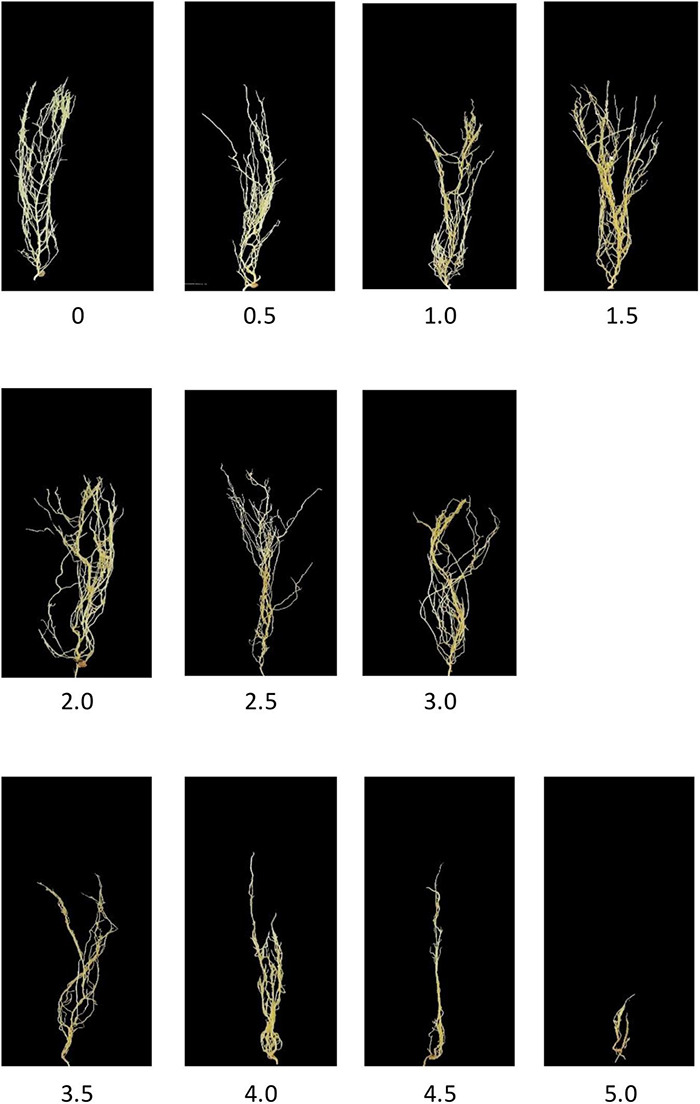
Aphanomyces root rot disease severity scale.

*Aphanomyces Root Rot Image Dataset* (Marzougui et al., 2019) contains up to 6,460 lentil images with root rot. ARR is a soil-borne disease that severely reduces lentil production. Based on the percentage of the brown discoloration area of the root and the softness of the hypocotyl (McGee et al., 2012), Marzougui et al. (2019) labeled the relative severity of all the root images using 0–5 disease scoring scale (McGee et al., 2012). For example, A score of 0 means that there are no obvious symptoms and good resistance to root rot; 1.5 means that the root has 15–25% of partial discoloration lesions; 3.5 means that the entire root has completely turned brown, and the hypocotyl has some symptoms. Eleven representative images with scores from 0 to 5 are shown in Figure 4. Furthermore, Marzougui et al. (2020) proposed three subtypes of ARR based on the visual score to evaluate the Rot severity: (1) resistant subtype with score 0–1.5; (2) partially resistant with score 2–3; (3) susceptible subtype with score 3.5–5. In this study, we selected 950 representative images of ARR for experimental simulation.

### Simulation Design

Firstly, 1,797 digital images from MNIST were used to test the proposed method. Furthermore, we also compared the WDM-tSNE with the other five clustering strategies on MNIST. Secondly, a binary clustering test was further implemented on 400 strawberry and 400 cherry images to identify the diseased samples from the control. Thirdly, 300 tomato images were selected to test the clustering performance of our approach on three different diseases. Finally, we selected 950 ARR images to explore potential subtypes for the lentil invaded by Aphanomyces. We manually constructed balanced datasets and unbalanced datasets to evaluate if our approach is steady. The sample size for each dataset is presented in Supplementary File 1.

### Clustering Performance Evaluation

In this study, we defined three types of metrics to assess the clustering performance. (1) Silhouette Coefficient (SC) (Dinh et al., 2019); (2) Calinski-Harabasz Index (CHI) (Łukasik et al., 2016); (3) Davies-Bouldin Index (DBI) (Vergani and Binaghi, 2018).

Silhouette Coefficient was firstly proposed by Rousseeuw (1987), which considered both the degree of cohesion and separation to measure the clustering performance. The *SC* value of sample *j* can be calculated by Eq. (11):


(11) $$SC_{j}=\frac{C_{j}-S_{j}}{\max\left\{C_{j},S_{j}\right\}}$$

where *C*_*j*_ and *S*_*j*_ represent the degree of cohesion and separation, respectively. We can clearly see that good clustering means smaller *C*_*j*_ and larger *S*_*j*_.

Calinski-Harabasz Index is defined as the ratio of the between-clusters dispersion mean and the within-cluster dispersion. A larger CHI means that the clusters themselves are tighter and the cluster-clusters are more dispersed [Eq. (12)]:


(12) $$CH=\left[\frac{\sum_{k-1}^{K}n_{k}{||c_{k}-c||}^{2}}{K-1}\right]/\left[\frac{\sum_{k-1}^{K}\sum_{i-1}^{n_{k}}{||d_{i}-c_{k}||}^{2}}{N-K}\right]$$

In Eq. (12), *N* and *K* are the number of samples and clusters, respectively. The variables *n*_*k*_ and *c*_*k*_ are the no. of points and centroid of the *h*-th cluster respectively, *c* is the global centroid.

Davies-Bouldin Index measures the average similarity between clusters [Eq. (13)].


(13) $$DB=\frac{1}{k}\sum_{i=1}^{k}\max_{i\neq j}R_{ij}$$

In Eq. (13), *R*_*ij*_ denotes the similarity between each cluster *C*_*i*_ and its most similar one *C*_*j*_:


(14) $$R_{ij}=\frac{s_{i}+s_{j}}{d_{ij}}$$

*s*_*i*_ denotes the average distance between each point of cluster *i*. *d*_*ij*_ denotes the distance between cluster centroids *i* and *j*.

### Parameter Optimization

All the simulations were performed using Python with TensorFlow on Ubuntu 14.04 platform. The hardware setups are 2.30?GHz CPU and 4.00 GB RAM. CNN model is composed of 13 convolutional layers, and each layer uses a stacked 3 × 3 small convolution kernel to replace the large-size convolution kernel. After each convolutional layer, a 2 × 2 max pooling is used. In the WDM-tSNE model, the gradient descent strategy is used to optimize the cost function *C* [Formula (9)], and the momentum term α^(*t*)^ is introduced to reduce the number of iterations (*T*). When the value of the cost function reaches 95% of the previous time, it indicates that the best result has been obtained, and the iteration is stopped. If *T < 250*, we set α^(*t*)^ = 0.5; otherwise, α^(*t*)^ = 0.8. The initial learning rate is set to 100, which is updated by the adaptive learning algorithm after each iteration.

## Results

### Validation on Modified National Institute of Standards and Technology Dataset

**FIGURE 5:**
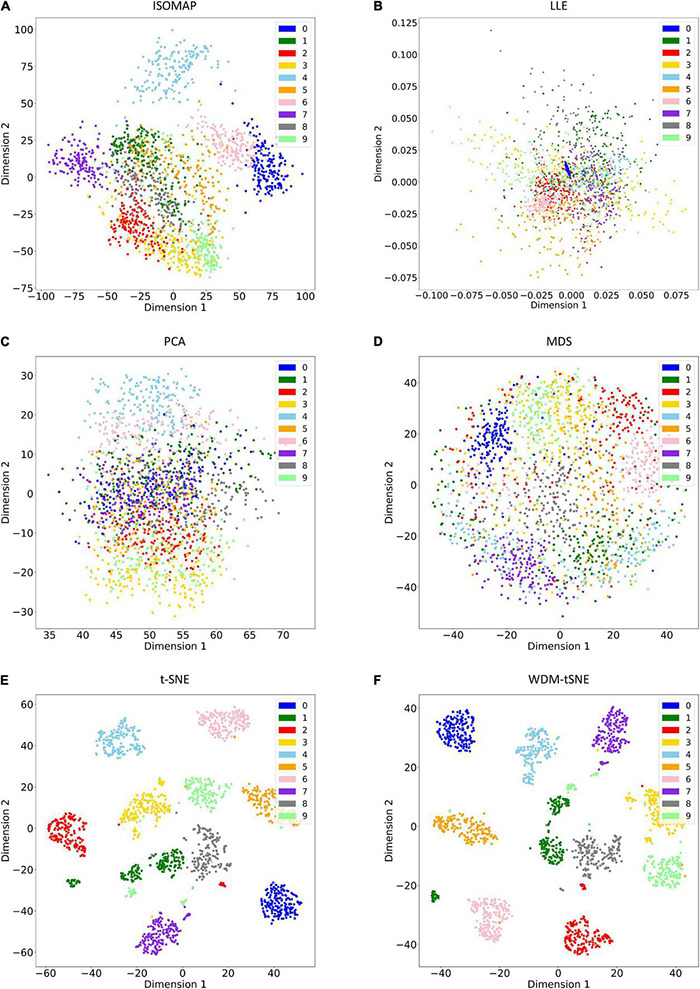
The plots for the MNIST dataset based on six dimensionality reduction approaches, including **(A)** Isomap, **(B)** LLE, **(C)** PCA, **(D)** MDS, **(E)** t-SNE, and **(F)** WDM-tSNE. MNIST, Modified National Institute of Standards and Technology; ISOMAP, Isometric Mapping; PCA, Principal Component Analysis; LLE, Locally Linear Embedding; MDS, Multidimensional Scaling; t-SNE, t-Distributed Stochastic Neighbor Embedding; WDM-tSNE, Weighted Distance Metric and the t-stochastic neighbor embedding algorithm.

As a golden-standard image dataset, MNIST was firstly tested by our method. A total 1,797 digital images were imported to the CNN module and converted to a 1,797 × 64 matrix. Moreover, all the 1,797 samples in a 64-D space were then projected to 2D space by six dimensionality reduction approaches, namely, ISOMAP (Isometric Mapping), PCA (Principal Component Analysis), LLE (Locally Linear Embedding), MDS (Multidimensional Scaling), t-SNE (t-Distributed Stochastic Neighbor Embedding), and the proposed WDM-tSNE (Figure 5). From Figure 5, we found that LLE and PCA obtained the worst performance of dimensionality reduction as the 10 types of digital images in 2D space cannot be separated at all. ISOMAP and MDS work better rather than the first two, but the boundaries of inter-clusters are still blurred. In contrast, t-SNE and WDM-tSNE are significantly better than the previous four methods. Particularly, multiple evaluation metrics indicates that the WDM-tSNE strategy obtained higher clustering accuracy on MNIST superior to the standard t-SNE (Supplementary Table 1). For the geometric distribution of the samples in 2D space, WDM-tSNE can obtain better partitions of clusters (Supplementary Table 1).

### The Proposed Model Works Well for Disease Diagnosis

**FIGURE 6:**
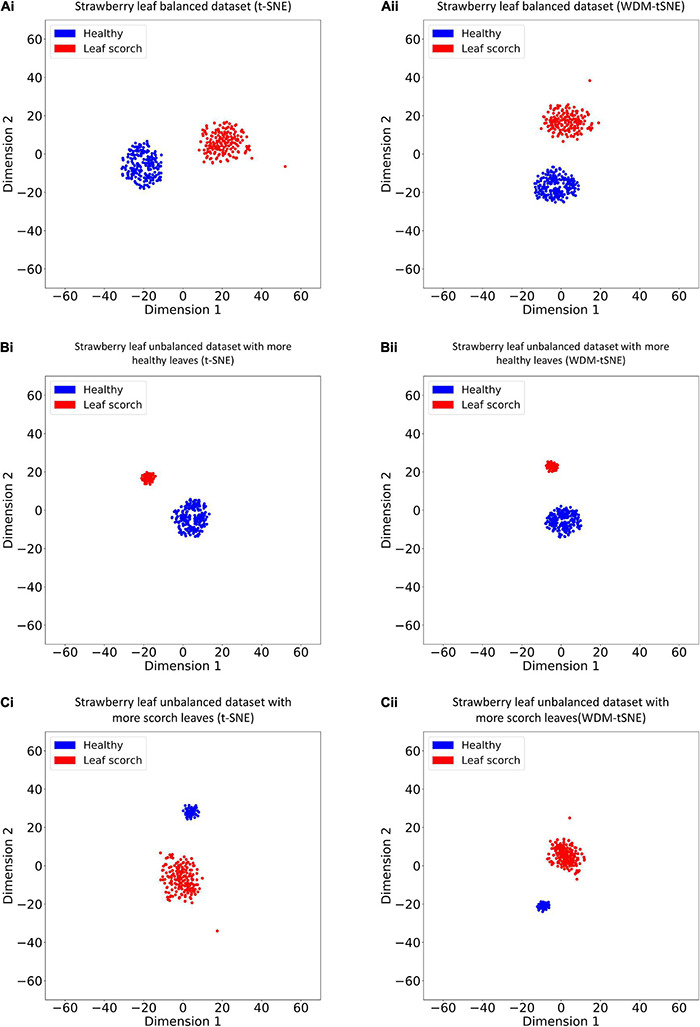
The plots for the **(A)** balanced or **(B–C)** unbalanced datasets of strawberry leaf scorch based on (i) t-SNE and (ii) WDM-tSNE. WDM-tSNE, Weighted Distance Metric and the t-stochastic neighbor embedding algorithm.

**FIGURE 7:**
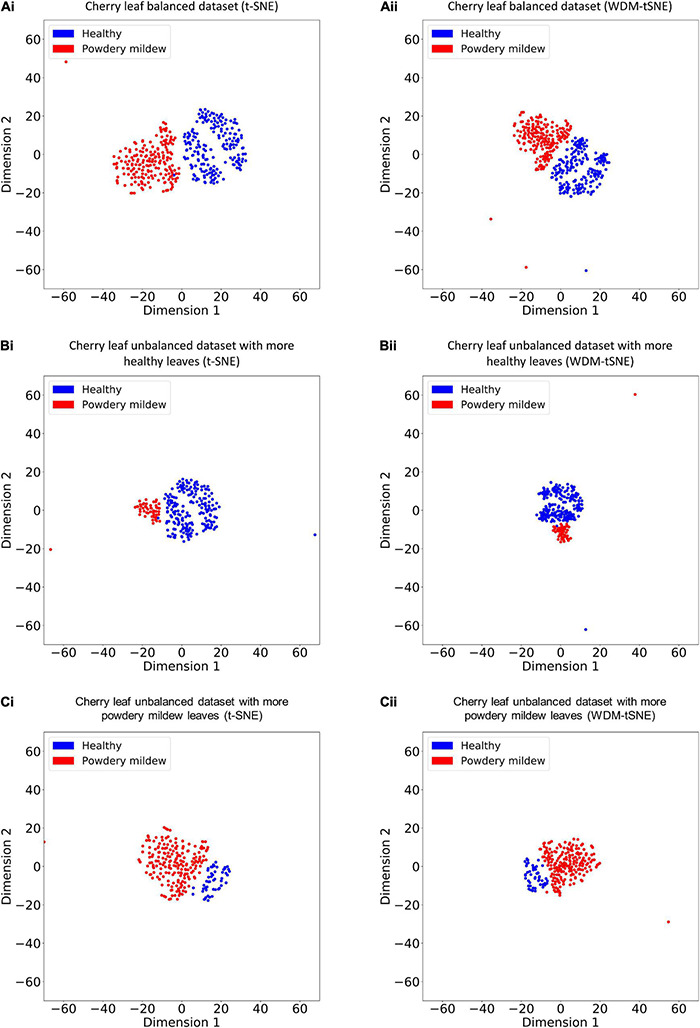
The plots for the **(A)** balanced and **(B–C)** unbalanced datasets of the cherry leaf with powdery mildew based on (i) t-SNE and (ii) WDM-tSNE. WDM-tSNE, Weighted Distance Metric and the t-stochastic neighbor embedding algorithm.

**FIGURE 8:**
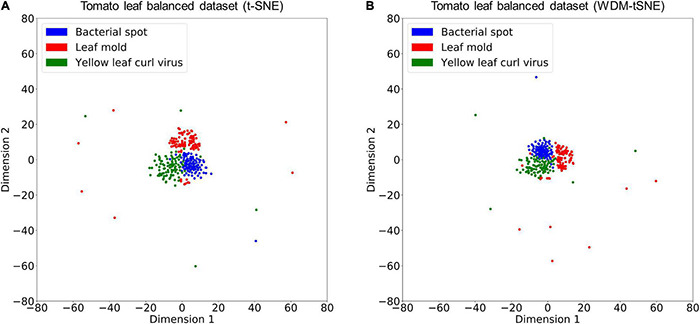
The plots for the balanced datasets of three tomato leaf diseases based on **(A)** t-SNE and **(B)** WDM-tSNE. WDM-tSNE, Weighted Distance Metric and the t-stochastic neighbor embedding algorithm.

We then applied our method on 400 strawberry images with leaf scorch. Figure 6 shows that the scorched leaf images can be easily identified from the healthy samples. Both balanced and unbalanced datasets revealed that the clustering performance is steady. Table 1 indicates that WDM-tSNE provides better clustering performance rather than t-SNE. Similarly, we also tested our approach on 400 cherry leaf images with powdery mildew. WCD-tSNE not only makes the samples in the same cluster more concentrated, but also guarantees the distance between different clusters is as far away as possible (Figure 7). Compared with t-SNE, WDM-tSNE has a better clustering effect (Table 2). In addition to the binary-clustering, we also tested the multi-clustering situation on the leaf images of diseased tomato. Figure 8 reveals that three distinct leaf diseases on tomatoes can be clearly identified (Table 3). Taken above together, we suggest that the proposed framework is an effective tool for identifying plant disease with high accuracy.

**TABLE 1:** The performance of WDM-tSNE on the multiple datasets of strawberry.

	Balanced dataset		Unbalanced dataset with more healthy leaves		Unbalanced dataset with more scorch leaves	
	t-SNE	WDM-tSNE	t-SNE	WDM-tSNE	t-SNE	WDM-tSNE
Silhouette coefficient	0.723	0.729	0.755	0.788	0.725	0.799
Calinski-Harabasz	2014.769	2106.024	1026.573	1353.780	734.780	1391.950
Davies-Bouldin Index	0.4070	0.399	0.271	0.233	0.302	0.218

*WDM-tSNE, Weighted Distance Metric and the t-stochastic neighbor embedding algorithm.*

**TABLE 2:** The performance of WDM-tSNE on the multiple datasets of cherry.

	Balanced dataset		Unbalanced dataset with more healthy leaves		Unbalanced dataset with more scorch leaves	
	t-SNE	WDM-tSNE	t-SNE	WDM-tSNE	t-SNE	WDM-tSNE
Silhouette coefficient	0.496	0.494	0.362	0.369	0.354	0.361
Calinski-Harabasz	511.877	540.454	81.172	103.808	119.424	131.842
Davies-Bouldin Index	0.773	0.764	0.969	0.841	0.836	0.829

*WDM-tSNE, Weighted Distance Metric and the t-stochastic neighbor embedding algorithm.*

**TABLE 3:** The performance of WDM-tSNE on the dataset of tomato disease.

	Balanced dataset	
	t-SNE	WDM-tSNE
Silhouette coefficient	0.263	0.273
Calinski-Harabasz	25.538	40.427
Davies-Bouldin Index	1.615	1.279

*WDM-tSNE, Weighted Distance Metric and the t-stochastic neighbor embedding algorithm.*

### The Proposed Model Works Well for Subtype Discovery

**FIGURE 9:**
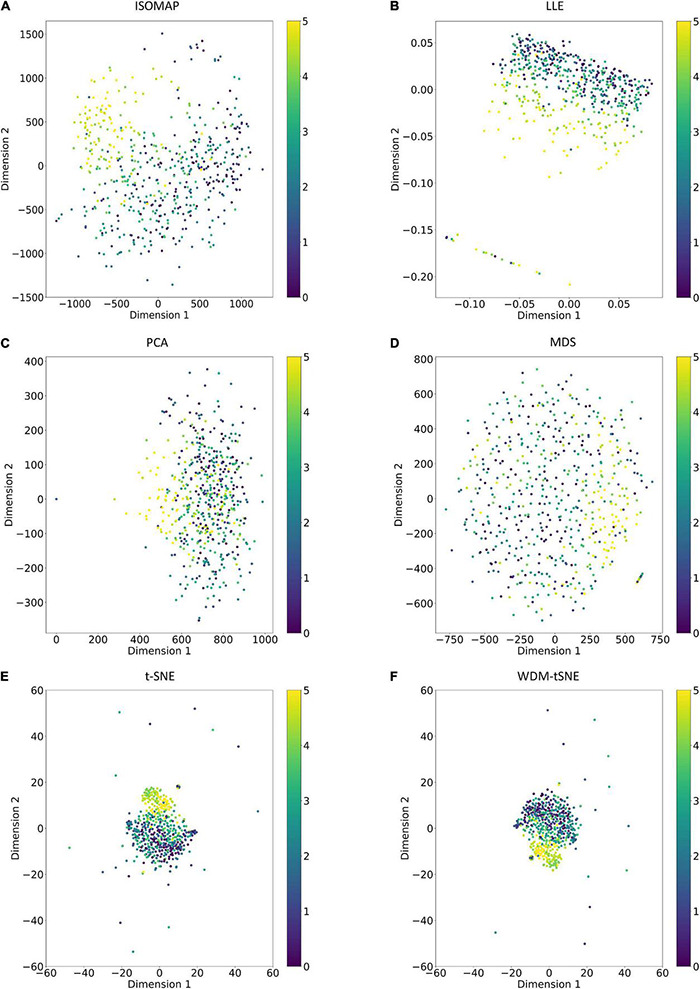
The plots for the balanced dataset of ARR based on six dimensionality reduction approaches, including **(A)** Isomap, **(B)** LLE, **(C)** PCA, **(D)** MDS, **(E)** t-SNE, and **(F)** WDM-tSNE. The samples with 11 rates were plotted. ARR, Aphanomyces Root Rot.

**FIGURE 10:**
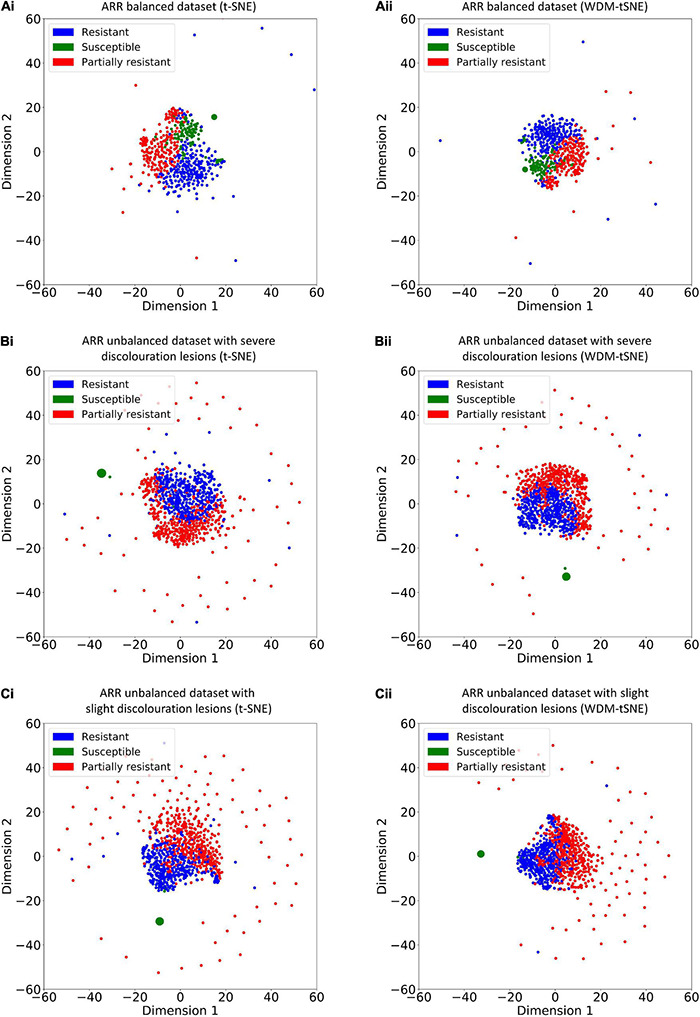
The plots for the **(A)** balanced and **(B–C)** unbalanced datasets of ARR are based on (i) t-SNE and (ii) WDM-tSNE. WDM-tSNE, Aphanomyces Root Rot; Weighted Distance Metric and the t-stochastic neighbor embedding algorithm; ARR, Aphanomyces Root Rot.

**FIGURE 11:**
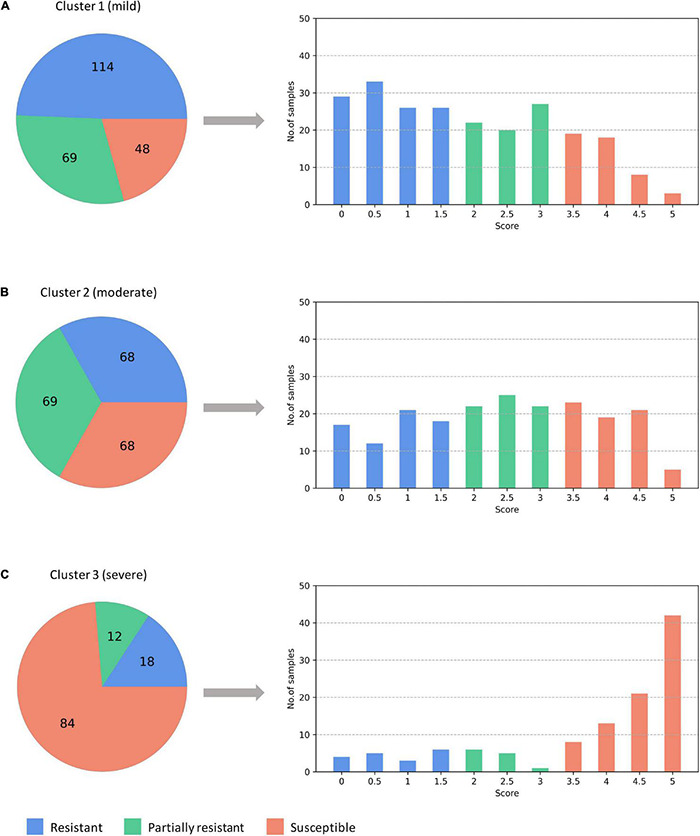
The predicted three subtypes of ARR: **(A)** mild; **(B)** moderate; **(C)** severe. The numbers denote how many samples are assigned to one of the subtypes. ARR, Aphanomyces Root Rot.

Different from the experiments shown above, we further applied our model on 950 lentil root images infected by Aphanomyces euteiches to identify the underlying subtypes associate with Aphanomyces resistance. Firstly, 550 representative images (balanced dataset) of ARR with 11 rates of severity were projected to 2D space through six machine-learning approaches (Figure 9). Figures 9E,F shows that both t-SNE and WDM-tSNE can uncover the disease trajectory of all the samples from mild to severe. Secondly, we selected 550 images (50 samples for each rate) to test if WDM-tSNE has the ability to reveal the underlying subtypes of the plant samples with the same disease. Figure 10 shows that three clusters are obviously detected from balanced and unbalanced datasets. The clustering performance of WDM-tSNE is superior to t-SNE (Table 4). In the balanced dataset with 550 samples, 231 were predicted as a mild subtype with an average score of 1.93, 205 were predicted as a partially moderate subtype (average score: 2.45), and 114 were marked as a severe subtype (average score: 3.74) (Figure 11). Figure 11 also suggests that the samples with serious symptoms can be easily detected (cluster 3). However, the visual score based on the percentage of discolored lesions on the entire root system defined by Marzougui et al. may cause bias when dividing mild and moderate samples. Therefore, the data annotations based on expert knowledge are also one of the factors that affect the accuracy of the algorithm.

**TABLE 4:** The performance of WDM-tSNE on the multiple datasets of lentil.

	Balanced dataset		Unbalanced dataset with severe discoloration lesions		Unbalanced dataset with slight discoloration lesions	
	t-SNE	WDM-tSNE	t-SNE	WDM-tSNE	t-SNE	WDM-tSNE
Silhouette coefficient	0.214	0.232	0.192	0.207	0.189	0.225
Calinski-Harabasz	130.182	165.652	163.077	182.195	161.421	237.701
Davies-Bouldin Index	1.279	1.147	1.799	1.667	1.402	1.235

*WDM-tSNE, Weighted Distance Metric and the t-stochastic neighbor embedding algorithm.*

## Discussion

Plant diseases are not only a threat to food security on a global scale, but also cause disastrous consequences for smallholder farmers whose livelihoods depend on healthy crops (Mohanty et al., 2016). Identifying a disease correctly when it first appears is a crucial step for efficient disease management. Various efforts have been developed to prevent the loss of the plant due to diseases. For computer-vision-based plant diseases detection, conventional image processing or manual design of features plus classifiers are often used (Tsaftaris et al., 2016). This kind of method usually makes use of the different properties of plant disease to design the imaging scheme and chooses appropriate light sources and shooting angles, which is helpful to obtain images with uniform illumination. In the real complex natural environment, plant diseases detection is faced with many challenges, such as the small differences between the lesion area and the background, low contrast, large variations in the scale of the lesion area and various types, and a lot of noise in the lesion image (Liu and Wang, 2021). In addition, over-depend on expert knowledge to manually design the features of diseased plant often limits the generalization. In recent years, DL methods are widely used in various CV tasks for plant disease diagnosis. The most challenges of DL-based strategies include small sample size problem, fine-grained identification of small-size lesions in the early stage, and the performance under the influence of illumination and occlusion (Liu and Wang, 2021).

In this study, we proposed a computational framework for both plant disease identification and severity estimation (Figure 1). Firstly, we designed a CNN network structure as a feature extractor to obtain the image features of lesion regions of a diseased plant. The input original images are not required with a fixed size, which avoid the impacts of image distortion or geometric distortion on feature extraction. Secondly, a dimension reduction strategy, WDM-tSNE, was developed for the imaging clustering tasks by improving the t-SNE with WDM. WDM-tSNE successfully realized the efficient clustering of high-dimensional samples in low-dimensional space.

To validate the effectiveness, we applied the proposed model on a bunch of plant image datasets. The experimental results revealed that our method not only identifies multiple distinct diseases of the same plant but also estimates the severity of the same disease. Figures 5, 6 indicate that our model is able to distinguish multiple diseases in a low-dimensional space. Figures 7, 8 show that the diseased samples can be easily identified from the health samples. From Figure 9, we concluded that the proposed method can be used for subtype discovery or severity estimation from the same disease (ARR). The 10-fold cross-validation on the ARR dataset revealed that our model is robust (Supplementary Table 2). Furthermore, we applied our model on three small-scale datasets for cherry, strawberry, and tomato. The sample size of each class is only 50. Our analyses show that our model works well on small-scale image datasets (Supplementary Figure 1 and Supplementary Table 3).

Considering the fact that the class imbalance may impact the clustering performance, we constructed multiple balanced and unbalanced datasets for ARR (lentil), cherry, and strawberry (Supplementary File 1). Regardless of binary-class or multi-class, WDM-tSNE shows better clustering performance than t-SNE (Tables 1–4). It indicates that the sample variation does not affect the performance of our method.

The proposed WDM-tSNE outperformed other approaches. After extracting the features from images through the CNN module, we compared the clustering performance of WDM-tSNE with the other five dimension-reduction algorithms. Figures 5, 9 proved that WDM-tSNE is not only significantly better than ISOMAP, LLE, PCA, and MDS, but also prior to tSNE.

Recent advances in genomics technologies have greatly accelerated the progress in plant science (Varshney et al., 2021). There are some studies to link phenotypic data to genomic data for discovering the responsible genes or mutations that contributed to plant disease progression (Bolger et al., 2019). Particularly, the systems biology approaches developed by integrating multi-omics data will allow us to identify potential targets and predict new therapeutic strategies (Di Silvestre et al., 2018).

There are several limitations of our current method. Firstly, the features extracted from the plant images by the CNN module are non-semantic, thus, it is hard to interpretable for disease diagnosis and management. Secondly, the current approach only focused on a single disease for each cluster of the image but did not pay attention to the images of plants suffering from multiple diseases. Thirdly, we have not applied the current model on the high-throughput phenotypic images obtained from real natural environments. Finally, we cannot guarantee the clustering performance on the image samples of diseased plants whose severity is manually labeled by different experts.

## Conclusion

This paper proposes a novel computational framework for plant disease identification and subtype discovery from phenomics data. Our proposed method has achieved high accuracy and good generalization ability in all four public datasets than other deep embedding clustering of images, e.g., t-SNE, ISOMAP, etc.

Specifically, our method does not depend on prior knowledge. Moreover, the size of input images is also unlimited. As a novel embedding strategy, WDM-tSNE provides the perfect clustering performance rather than other methods. The samples in 2D space present great distributions after space embedding, which is significant to reveal the underlying patterns and trajectory of plant disease.

In the future, we will further explore the association between the environmental parameters (climate, hydrology, and soil, etc.) and plant disease evolution.

## Data Availability Statement

All the raw images involved in this study can be accessed through the links: https://xf-data-bucket.oss-cn-hangzhou.aliyuncs.com/data.rar. Source code is available at GitHub: https://github.com/JakeJiUThealth/WDM1.0. The original contributions presented in the study are included in the article/Supplementary Material, further inquiries can be directed to the corresponding author.

## Author Contributions

ZJ, FX, and XX designed the project. FX and XX analyzed the data. ZJ performed the mathematical modeling and optimization. ZJ, FX, ZW, SJ, and KY discussed the results. ZJ and FX wrote the manuscript. All authors contributed to the article and approved the submitted version.

## Conflict of Interest

The authors declare that the research was conducted in the absence of any commercial or financial relationships that could be construed as a potential conflict of interest.

## Publisher’s Note

All claims expressed in this article are solely those of the authors and do not necessarily represent those of their affiliated organizations, or those of the publisher, the editors and the reviewers. Any product that may be evaluated in this article, or claim that may be made by its manufacturer, is not guaranteed or endorsed by the publisher.

## Supplementary Material

Supplementary Figure 1 The plots for the small-scale balanced datasets are based on t-SNE and WDM-tSNE. WDM-tSNE, Weighted Distance Metric and the t-stochastic neighbor embedding algorithm.

Supplementary Table 1 Clustering performance on MNIST dataset. MNIST, Modified National Institute of Standards and Technology.

Supplementary Table 2 A 10-fold cross-validation on the balanced dataset of ARR samples. ARR, Aphanomyces Root Rot.

Supplementary Table 3 Clustering performance on three small-scale datasets. The sample size of each class is 50.

Supplementary File 1 The sample assignment for each dataset involved in this study.
