# Supplementary figures and images for: A Novel Computational Framework for Precision Diagnosis and Subtype Discovery of Plant With Lesion
Source: Front Plant Sci. 2022 Jan 3;12:789630. doi: 10.3389/fpls.2021.789630 (PMC8761810; doi:10.3389/fpls.2021.789630)

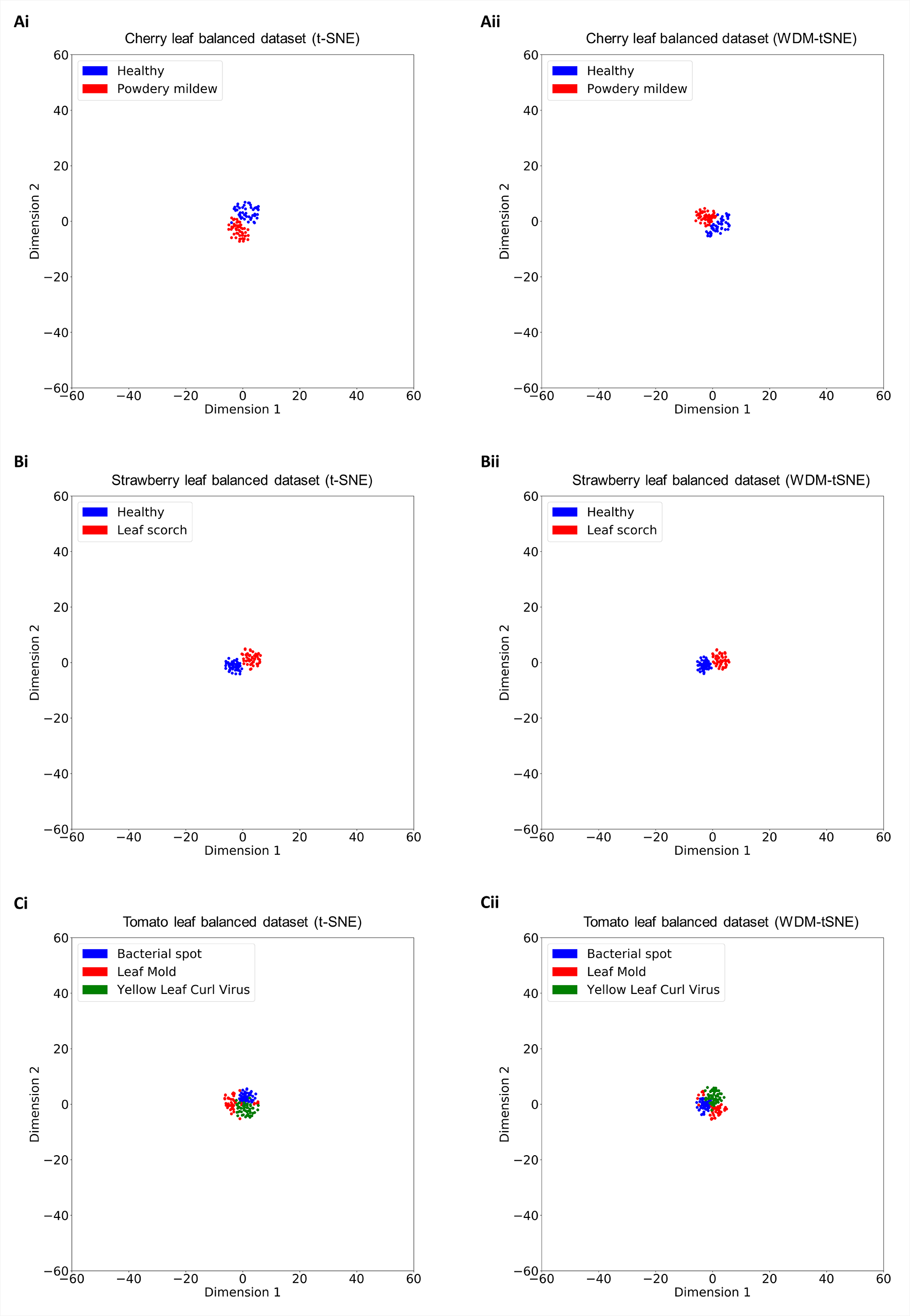

Supplement: Supplementary Figure 1 — The plots for the small-scale balanced datasets are based on t-SNE and WDM-tSNE. WDM-tSNE, Weighted Distance Metric and the t-stochastic neighbor embedding algorithm. [file Image_1.TIF]
