# Supplementary material for: A Novel Computational Framework for Precision Diagnosis and Subtype Discovery of Plant With Lesion
Source: Front Plant Sci. 2022 Jan 3;12:789630. doi: 10.3389/fpls.2021.789630 (PMC8761810; doi:10.3389/fpls.2021.789630)
Supplement: Supplementary Table 1 — Clustering performance on MNIST dataset. MNIST, Modified National Institute of Standards and Technology. [file Table_1.DOCX]

**Supplementary Tables**

**Table S1**. Clustering performance on MNIST dataset**.**

| **No.** | **Evaluation metric** | **t-SNE** | **WCD-tSNE** |
| --- | --- | --- | --- |
| 1 | ARI^[1]^ | 0.892 | 0.901 |
| 2 | AMI^[2]^ | 0.914 | 0.920 |
| 3 | Homogeneity^[3]^ | 0.915 | 0.921 |
| 4 | Completeness^[3]^ | 0.916 | 0.922 |
| 5 | V-measure^[3]^ | 0.915 | 0.921 |
| 6 | Fowlkes mallows score^[4]^ | 0.903 | 0.911 |
| 7 | Precision^[5]^ | 94.97% | 95.27% |
| 8 | Recall^[6]^ | 94.71% | 95.10% |
| 9 | F1 score^[7]^ | 94.68% | 95.05% |
| 10 | Silhouette Coefficient | 0.166 | 0.169 |
| 11 | Calinski-Harabasz index | 151.003 | 151.161 |
| 12 | Davies-Bouldin Index | 2.166 | 2.135 |

**Table S2**. 10-fold cross validation on the balanced dataset of ARR samples.

| **Evaluation metrics** | **t-SNE** | **WDM-tSNE** |
| --- | --- | --- |
| Silhouette Coefficient | 0.167$\pm$0.03 | 0.207$\pm$0.04 |
| Calinski-Harabasz index | 99.685‬$\pm$33.85 | 112.052$\pm$40.18 |
| Davies-Bouldin Index | 1.262$\pm$0.17 | 1.202$\pm$0.17 |

**Table S3**. Clustering performance on three small-scale datasets. The sample size of each class is 50.

| **Evaluation metrics** | **Cherry powdery mildew** | | **Leaf scorch of strawberry** | | **Leaf diseases on tomato** | |
| --- | --- | --- | --- | --- | --- | --- |
|  | t-SNE | WDM-tSNE | t-SNE | WDM-tSNE | t-SNE | WDM-tSNE |
| Silhouette Coefficient | 0.446 | 0.481 | 0.573 | 0.574 | 0.252 | 0.223 |
| Calinski-Harabasz index | 113.186 | 147.786 | 207.960 | 212.002 | 9.579 | 10.988 |
| Davies-Bouldin Index | 0.857 | 0.759 | 0.624 | 0.617 | 1.877 | 1.276 |

**References**

1. Steinley, D., *Properties of the Hubert-Arable Adjusted Rand Index.* Psychological Methods, 2004. **9**(3): p. 386–396.

2. Nguyen Xuan Vinh, J.E., James Bailey, , *Information Theoretic Measures for Clusterings Comparison: Variants, Properties, Normalization and Correction for Chance.* Journal of Machine Learning Research, 2010. **11**: p. 2837-2854.

3. Andrew Rosenberg, J.H., *V-Measure: A conditional entropy-based external cluster evaluation measure.* Proceedings of the 2007 Joint Conference on Empirical Methods in Natural Language Processing and Computational Natural Language Learning, 2007: p. 410-420.

4. Fowlkes EB , M.C., *A method for comparing two hierarchical clusterings.* Journal of the American Statistical Association, 1983. **78**(383): p. 553-569.

5. Tao Liu, S.L., Zheng Chen, Wei-Ying Ma *An Evaluation on Feature Selection for Text Clustering.* Proceedings of the Twentieth International Conference on Machine Learning (ICML-2003), 2003: p. 1-8.

6. Lailil Muflikhah, B.B., *Document Clustering Using Concept Space and Cosine Similarity Measurement.* 2009 International Conference on Computer Technology and Development, 2009: p. 1-5.

7. Chicco, D. and G. Jurman, *The advantages of the Matthews correlation coefficient (MCC) over F1 score and accuracy in binary classification evaluation.* BMC Genomics, 2020. **21**(1): p. 6.
